# Structural and Dynamical Insights on HLA-DR2 Complexes That Confer Susceptibility to Multiple Sclerosis in Sardinia: A Molecular Dynamics Simulation Study

**DOI:** 10.1371/journal.pone.0059711

**Published:** 2013-03-26

**Authors:** Amit Kumar, Eleonora Cocco, Luigi Atzori, Maria Giovanna Marrosu, Enrico Pieroni

**Affiliations:** 1 Multiple Sclerosis Center, Department of Public Health and Clinical and Molecular Medicine, University of Cagliari, Cagliari, Italy; 2 CRS4 Science and Technology Park Polaris, Bio-Engineering Group, Piscina Manna, Pula (CA) Italy; 3 Department of Biomedical Sciences, Oncology and Molecular Pathology Unit, University of Cagliari, Cagliari, Italy; University of Bologna & Italian Institute of Technology, Italy

## Abstract

Sardinia is a major Island in the Mediterranean with a high incidence of multiple sclerosis, a chronic autoimmune inflammatory disease of the central nervous system. Disease susceptibility in Sardinian population has been associated with five alleles of major histocompatibility complex (MHC) class II DRB1 gene. We performed 120 ns of molecular dynamics simulation on one predisposing and one protective alleles, unbound and in complex with the two relevant peptides: Myelin Basic Protein and Epstein Barr Virus derived peptide. In particular we focused on the MHC peptide binding groove dynamics. The predisposing allele was found to form a stable complex with both the peptides, while the protective allele displayed stability only when bound with myelin peptide. The local flexibility of the MHC was probed dividing the binding groove into four compartments covering the well known peptide anchoring pockets. The predisposing allele in the first half cleft exhibits a narrower and more rigid groove conformation in the presence of myelin peptide. The protective allele shows a similar behavior, while in the second half cleft it displays a narrower and more flexible groove conformation in the presence of viral peptide. We further characterized these dynamical differences by evaluating H-bonds, hydrophobic and stacking interaction networks, finding striking similarities with super-type patterns emerging in other autoimmune diseases. The protective allele shows a defined preferential binding to myelin peptide, as confirmed by binding free energy calculations. All together, we believe the presented molecular analysis could help to design experimental assays, supports the molecular mimicry hypothesis and suggests that propensity to multiple sclerosis in Sardinia could be partly linked to distinct peptide-MHC interaction and binding characteristics of the antigen presentation mechanism.

## Introduction

**Figure 1:**
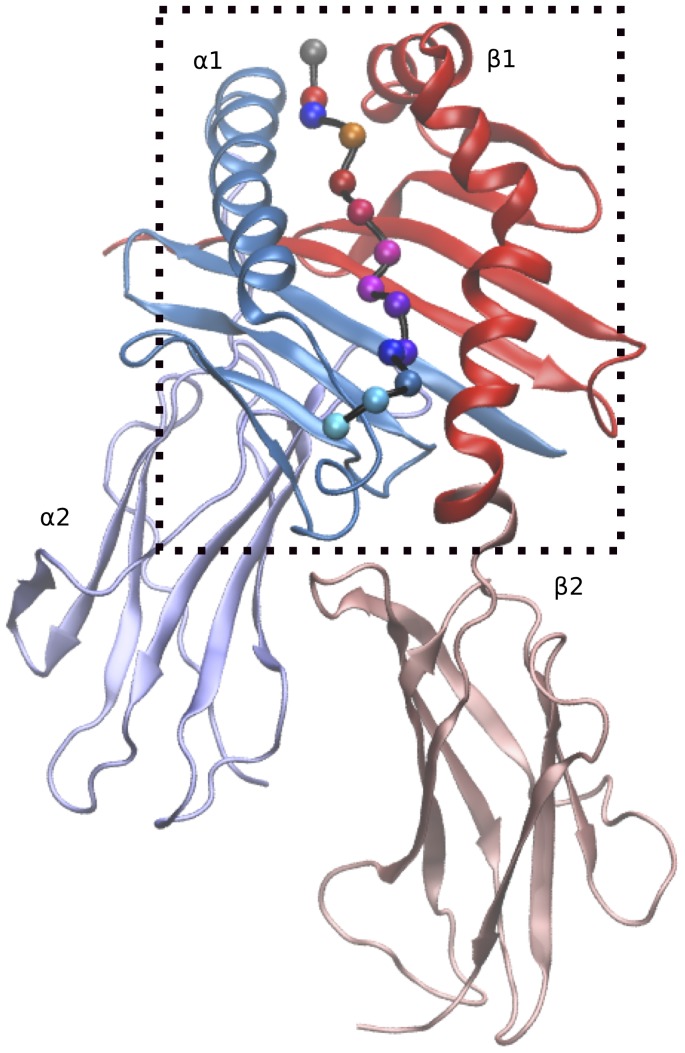
The peptide-MHC complex. The two chains A and B are shown in cartoon representation. The binding groove, formed by domain α1 and β1 of chain A and B, respectively, is boxed by dashed line in black. The MBP peptide is represented by ball and stick, with each ball corresponding to C-alpha atom, and its color representing the residue type.

Multiple Sclerosis (MS) is an autoimmune disease associated to inflammatory and degenerative processes in the central nervous system [1]. Human Leukocyte Antigen (HLA), and in particular HLA class II system has been identified as the main genetic determinant region linked to MS [2], specifically the major histocompatibility complex (MHC) class II DRB1 gene has been found to be strongly associated with MS susceptibility [3], [4]. HLA system class II molecules are membrane glycoproteins expressed on specialized antigen presenting cells that have a remarkable capacity to bind and present antigenic peptides, which is a critical component of the adaptive immune response to foreign pathogens [5]. The formed peptide-MHC class II complexes (Fig. 1) are then recognized by antigen specific T-cell receptors (TCR) leading to T-cell activation, differentiation, proliferation and finally to a specific immune response to pathogens [5], [6].

The aim of the current study is to analyze in detail the features of the peptide-MHC complex, particularly the local flexibility of the binding cleft and the emerging peptide interaction networks, because this information is an indirect measure of the TCR affinity. The three-stage connection between peptide-MHC dynamical features, T cell affinity and activation potency is quite complex [7], [8] but, importantly, the most accepted TCR binding models [9], [10], [11], [12], [13] are based on a reciprocal conformational plasticity of both TCR and peptide-MHC, thus requiring a certain degree of peptide-MHC flexibility for a successful TCR recognition and then a conformational adjustment upon TCR binding [14], [15]. Recently the issue was investigated by many groups, in particular some authors [16], [17] provided important evidences, both computational and experimental, supporting a direct link between MHC protein flexibility – ‘floppy state’ – and enhanced peptide loading capabilities, with or without the help of an ancillary peptide loading enhancer protein called DM. Other works evidenced a binding groove closure [18], [19], due to stabilizing hydrogen bond reducing solvent exposure in the unbound MHC protein or saturating peptide reserved sites. More recently, some authors observed that the characteristics of the peptide-MHC interaction with the TCR can partly represent the environment where the peptide was acquired, thus highlighting the importance to have a sufficiently prolonged – that is stable – peptide-MHC interaction capable to shape resistant and unique peptide-MHC surfaces [20]. On the experimental ground, peptide-MHC binding assays have been used to classify peptides as binders (IC_50_ ≤ 1000 nM) and non-binders (IC_50_ ≥1000 nM) [21]. Moreover, a vast number of computational methods have been applied to predict peptide-MHC binding, for instance based on either (i) scoring matrices on quantitative binding data [22] (ii) multiple peptide alignments [23] or (iii) qualitative structure activity relationship [24]. Recently, using the consensus approach, some authors [21] were able to demonstrate a better prediction performance for a large number of peptide-MHC class II complexes, compared to previous computational methodologies [22], [23], [24]. All the authors thus highlight the need of novel approaches that could capture distinct features of peptide-MHC interactions, to allow a successful prediction of the peptide-MHC binding.

In recent genetic studies [25] three HLA-DRB1 alleles (*15:01, *16:01, *15:02), belonging to the immunologically well characterized DR2 serological group, were analyzed to investigate their association to MS in Sardinian population. The present study focuses on the molecular characteristics of two of these DR2 alleles, one predisposing (*15:01) and one protective (*16:01), as an essential frame to understand allele resistance and susceptibility characteristics that can be eventually generalized to wider allele groups. Moreover, the selected alleles have a relevance in both North-Europe and Sardinia [2], [26]. More precisely, *15:01 is the most frequent and associated allele in Caucasian population, while to confirm the rather peculiar Sardinian genetic background, *15:01 allele frequency was found to be only 1.5% in Sardinian MS patients [27]. On the other hand *16:01 allele is associated to MS mainly in Sardinia, with frequency of 19.1% in patients.

**Figure 2:**
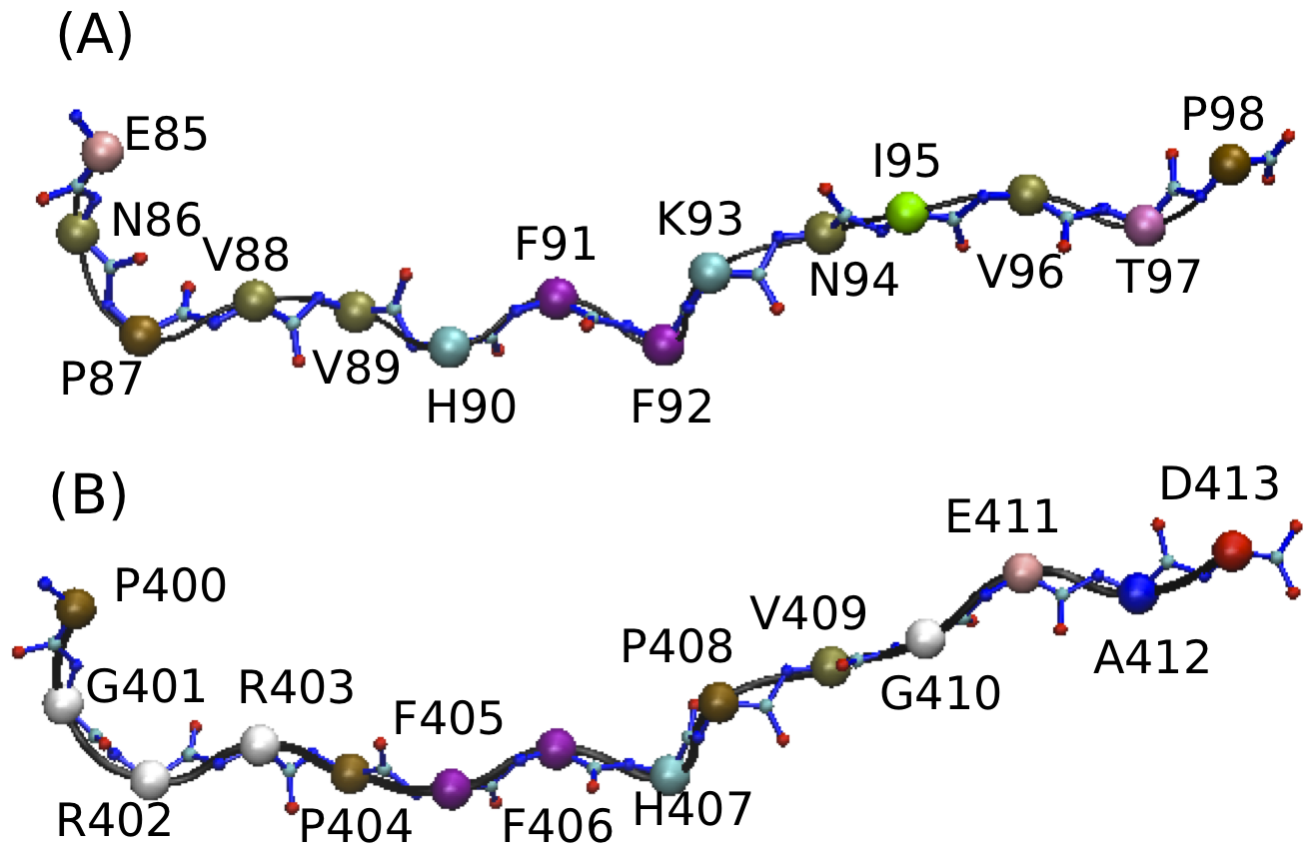
Peptide structure inside binding groove. (A) MBP 85–98 epitope, (B) EBNA-1 400–413 epitope.

MS is at present considered a complex disease, associated to an interplay between genetic and environmental conditions [28], [29], [30]. Myelin Basic protein (MBP) peptide, derived from myelin sheaths surrounding axons, is known to be one of the auto antigens important in the pathogenesis of MS [31], particularly epitope MBP 85–98 (Fig. 2A). As a characteristic environmental factor, the association of Epstein Barr Virus (EBV) with MS has been substantiated recently with the fact that EBNA-1 (Fig. 2B), a specific virus protein, is suggested as one of the most relevant non-self antigen candidate to induce MS [28], [32].

In case of a functional and molecular similarity – mimicry – between the self (MBP) and non-self (EBNA-1) peptide in complex with MHC, it may happen that an auto-reactive T cell could be triggered by a non-self peptide [33] **.** After peripheral activation, the T lymphocytes could then migrate to the central nervous system, where they might be reactivated (particularly by dendritic cells, astrocytes and microglia) because of cross-reactivity with respect to MBP, in a given inflammatory milieux and thus initiate or sustain an immune self-response. The overall process thus results in loosing immune homeostasis and boosting reactions against functionally important self-proteins, particularly damaging myelin in MS [34].

**Figure 3:**
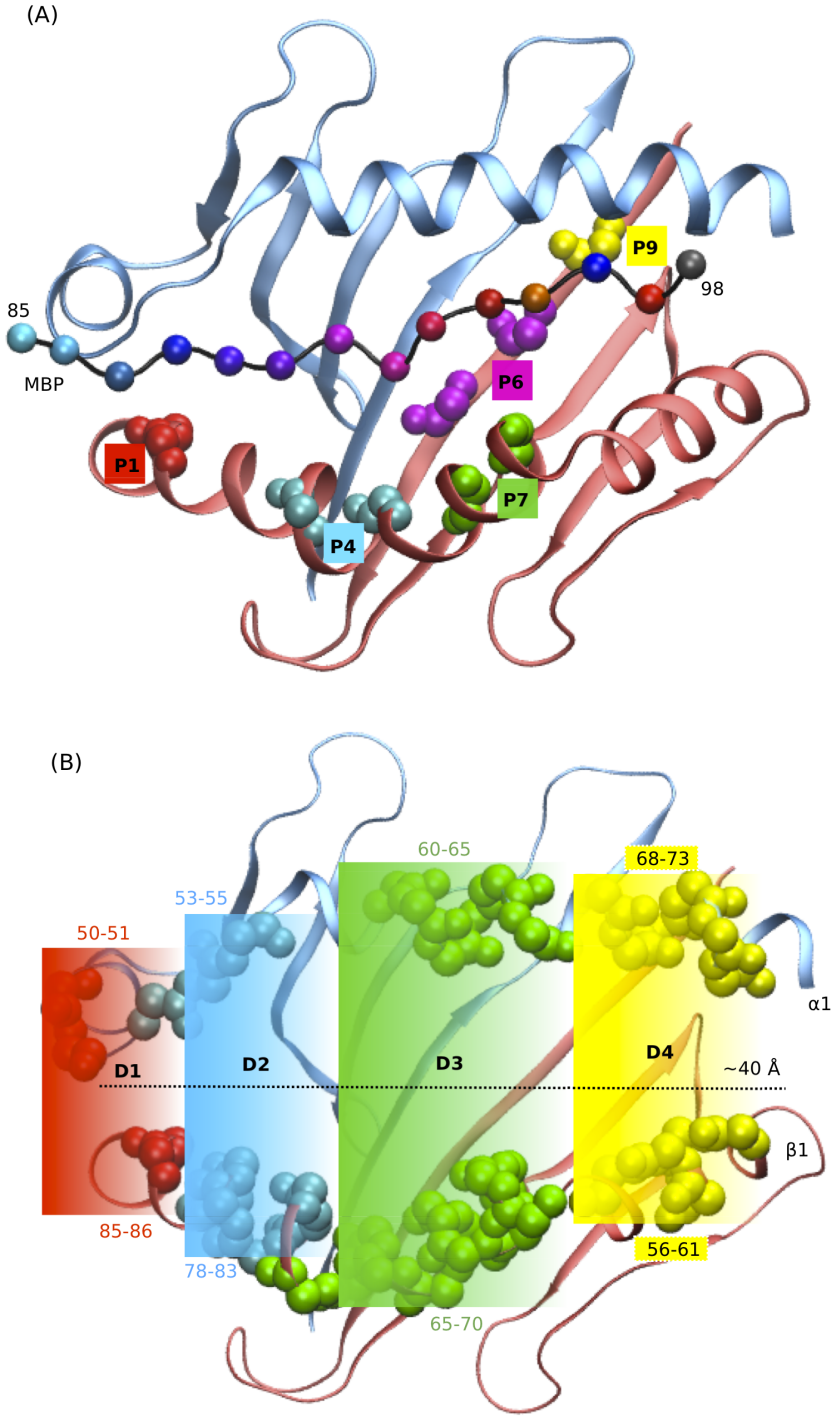
Pockets and four compartments of MHC class II binding groove. (A) The MHC cleft is shown using cartoon representation. The backbone of MBP peptide is shown in cartoon and the C-alpha atoms in sphere representations. The residues forming the pockets are shown in Van der Waals representation. (B) Region D1 (in red), D2 (blue), D3 (green) and D4 (yellow).

X-ray structures for some peptide-MHC class II complexes have been solved at high resolution [35], [36], [37], [38], [39], [40], thus providing useful structural insights into the interaction of the peptide and protein inside the complex. As evidenced by these findings, the three dimensional structure of MHC class II molecule is formed by two chains, chain A and chain B, with each chain formed by two domains, as shown in Fig. 1. The MHC class II binding groove for the peptide is formed as an interchain dimer of α1 and β1 domains of chain A and chain B respectively [41]. Furthermore, structural characterization of the peptide-MHC complex led to the identification of specific anchor residues in the MHC binding groove and the corresponding preferred residue profiles on the peptide. The peptide residue preferences are generally localized in peptide residue position 1, 4, 6 and 9, and vary for different HLA variants and alleles [42], [43], [44]. Recent theoretical studies have used variations in the electrostatic landscapes of MHC class II binding groove to distinguish the pockets' amino acids with a specific anchoring (Pocket 1 and 4) or recognition (Pockets 4 and 7) property (Fig. 3A) [45]. Moreover, preferences in peptide-binding shown by different HLA-DRB1 allele variants, suggest also a relevant role of the residues surrounding the pockets in disease susceptibility [36]. For instance, recent structural analysis performed for MS associated DR2 alleles in Sardinian population revealed polymorphism at position 86, belonging to pocket 1, as a relevant aspect characterizing the predisposing and protective alleles [25]. Interestingly, in another study carried out in Scandinavian patients suffering from primary sclerosing cholangitis, an autoimmune disease, residue 37 (pocket 9) and residue 86 (pocket 1) were found to distinguish predisposing (DRB1*13:01) and protective (DRB1*13:02) alleles [46]. Furthermore, structure modeling studies have shown interactions between pocket 6 and pocket 9 to influence binding preferences for pocket 9 of DRB1*09:01 allele, thus suggesting the importance of cooperative effects during the peptide binding process [47].

Molecular dynamics (MD) simulation is a powerful technique, which provides a high resolution dynamical picture (contrary to static representation provided by crystallography) and has been extensively used to model different biological relevant molecular systems [48], [49], [50]. Previous MD simulations have been performed to investigate changes in conformational dynamics of MHC-class II binding cleft, when free or complexed with a peptide [19], [51], [52], and on single amino-acid mutation of the peptide or MHC [8].

In the present study, we have performed long MD simulation run of 120 ns to investigate the structural and dynamical differences between two DR2 alleles: the predisposing DRA1*01:01_DRB1*15:01 and the protective DRA1*01:01_DRB1*16:01, when (i) unbound and complexed with (ii) myelin basic protein MBP 85–98 peptide (Fig. 2A), or (iii) Epstein Barr Virus protein EBNA-1 400–413 peptide (Fig. 2B). In particular, with the aim of studying peptide-MHC flexibility, following a recent approach [16], we divided the MHC binding groove in four compartments, corresponding to the influence region of the pockets 1, 4, 7 and 9. We focus on MHC class II peptide binding site region to characterize and emphasize the similarities and differences in binding properties between the two alleles by (a) calculating root mean square deviation (RMSD) and configurational entropy of the binding site residues, (b) evaluating the distance variation in four compartments of binding site (Fig. 3B), (c) evaluating the diverse nature of interactions between the binding site residues and the peptide, (d) estimating binding free energy values for the two peptides and finally (e) testing the binding by in-*silico* virtual single amino-acid substitution to alanine at six positions of the peptide and at four positions of MHC to determine their specificity and role in peptide-MHC interactions. As a whole, we present structural and dynamical information that allow to characterize the specific nature of the two MS relevant Sardinian alleles, by analyzing the peptide-MHC interaction patterns.

## Materials and Methods

### 1. From X-ray structure to Model system preparation

The starting structure for the predisposing allele DRB1*15:01 complexed with MBP 85–97 peptide was taken from protein data bank (PDB access code: 1BX2), while for the protective allele DRB1*16:01, due to unavailability of X-ray structure we performed homology modeling using the software MODELLER [53] with template DRB1*01:01 allele having a 94% sequence identity, complexed with CLIP peptide (PDB access code: 3PDO). The quality of the modeled structure was validated with the Ramachandran plot which showed 92.1% of residues in the most favored and 7.3% in the additional allowed region, with 0.6% in the generously allowed and none in disallowed region. These regions follow the convention as defined in PROCHECK [54] and the analysis was performed using Swiss model web interface [55]. Hydrogen atoms were added using the VMD software [56] for the protein-peptide complexes. The complexes were then centered in a rectangular water box and finally counter-ions were added to obtain a neutral system. The sequence for EBV EBNA-1 peptide 400–413 was selected from EBNA-1 protein, which has been identified to be associated to MS, as confirmed in a recent work [32]. The model structure for DRB1*15:01-EBNA-1 peptide complex was constructed using the template structure of DRB1*1501-MBP and the web server MODPROPEP [57], and the modeled peptide in the binding cleft was further checked using AUTO-DOCK [58] while for DRB1*1601-EBNA-1 complex we used program AUTO-DOCK to dock the EBNA-1 peptide in the binding cleft of the allele.

### 2. Molecular Dynamics Simulations

We used NAMD software package [59] to perform all-atom molecular dynamics (MD) simulations on a 64 nodes cluster. The parameters for the MHC-peptide complex were assigned alternatively using CHARMM27 force-field (C27-FF) parameters [60] and AMBER-99 force-field (A99-FF) parameters [61]. The TIP3P parameters [62] were used for water molecules. Standard protonation states were assigned to all residues except for Asp 66 residue of chain A, which was protonated as done in a recent MD simulation study of peptide-HLA-DR3 complex [51], where the authors showed the dynamics of the binding groove to be highly dependent on assignment of protonation state of key residues. The simulations for each of the two alleles *15:01 and *16:01 was then performed in the unbound peptide case and with both auto-antigen and pathogen-derived peptides. We summarize in Table 1 the simulations performed on the resulting different systems. The energy of the molecular system was then minimized and the system was gradually heated to 310 K in steps of 30 K with positional constraints of 50 kcal/(mol Å^2^) on carbon alpha atoms for a simulation time of 0.2 ns. The positional constraints on the carbon alpha were then slowly released in steps of 10 kcal/(mol Å^2^) and after 0.3 ns of they were completely released. The molecular system was then equilibrated for a simulation time of 3 ns. Subsequently, all production run of 120 ns simulation time was performed at 310 K and 1 atm pressure [63]. The initial dimension of the simulation box edges were [77 75 96] Å, for a total system of ∼50.000 atoms. All bonds involving hydrogen atoms were constrained using SHAKE [64], which allowed using an integration time step of 2 fs. The long-range electrostatic interactions were evaluated using particle mesh Ewald [65] with a [96 96 96] Å grid dimension. We used a 10 Å cut-off radius for both Van der Waals and electrostatic interactions along with smooth particle mesh Ewald [65].

**Table 1:** Peptide-MHC class II simulation lengths.

	Predisposing		Protective	
	C27-FF	A99-FF	C27-FF	A99-FF
**MBP (85**–**98)**	120 ns	60 ns	120 ns	60 ns
**EBNA-1 (400**–**413)**	120 ns	60 ns	120 ns	60 ns
**No-peptide**	120 ns	–	120 ns	–

Summary of MHC-peptide complexes modeled with different force fields (C27-FF and A99-FF). MD simulation on free MHC system was done using only C27-FF.

### 3. MD simulation analysis

Root mean square deviation (RMSD) was calculated on carbon alpha atoms for the selected binding site residues using VMD software [56]. The peptide binding groove was divided into four compartments: D1 (including residues α 50–51 and β 85–86), D2 (α 53–55 and β 78–83), D3 (α 60–65 and β 65–70), and (iv) D4 (α 68–73 and β 56–61), as shown in Fig. 3B. The center of mass distance variation of heavy atoms between the residues of α and β chains was calculated for the 120 ns MD simulation trajectory for each of the system under investigation. The hydrogen bond (H-bond) between β-chain binding site residues and the peptide residues was calculated using VMD script using the Donor-Acceptor cutoff distance of 3.1 Å and cutoff angle of 130°. The aromatic stacking interaction between the binding site residues and the peptide residues was calculated using EUCB software [66] with the dihedral angle cutoff parameters between the planar/ring side chains of 30°, centroid distance cutoff between side chains of 5.0 Å, and a minimum duration of 20% of simulation time. We also used EUCB software to identify hydrophobic region (isolated from water molecules) between the β-chain of the binding site residues and the peptide.

### 4. Configurational Entropy and Binding Free Energy calculations

From the 120 ns dynamics trajectory for each of the complex we extracted 600 structures consisting of only binding site residues. On each of the extracted structure the configurational entropy was estimated using the quasi-harmonic analysis as suggested by Andricioaei and Karplus [67], evaluating the covariance matrices of atomic fluctuations of the binding site residues, by using a routine incorporated in CARMA software package [68].

The binding free energy for the peptide-MHC complex was calculated using solvated interaction energy (SIE) method [69] and using the SIETRAJ software package [70]. To do so, we performed MD simulations with amber99 force-field parameters, a prerequisite for SIETRAJ calculations. The SIE free energy value was calculated at time step of 20 ps. The SIE approach is an alternative to the commonly used molecular mechanics Poisson-Boltzmann surface area (MM-PBSA) [71] methodology, based on similar treatment on electrostatics and non-bonded interactions. The main approximation used in the former is the scaling of SIE free energy by an empirically determined parameter obtained by fitting a training set of 99 protein-ligand complexes, thus allowing a crude but effective treatment of entropy-enthalpy compensation. Virtual alanine substitution at six different positions of the peptide and four positions of MHC was also done using the SIETRAJ software [70] and SIE energy was calculated in each case. Furthermore, we can obtain the concentration of peptide required to bind 50% of MHC (IC_50_), using the peptide-MHC free energy value as follows: ▵G_(SIE_)≈k_B_T ln(IC_50_) [72], where k_B_ is the Boltzmann constant and T is the temperature.

## Results

### 1. The MHC class II binding groove is stable in the presence of the peptide

**Figure 4:**
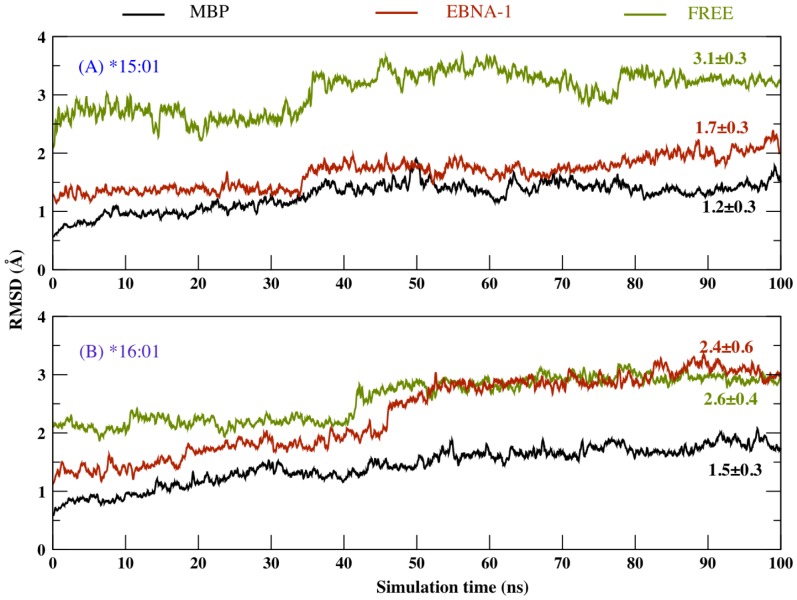
RMSD time plot. C-alpha root mean square deviation variation with respect to initial frame obtained during long MD simulations, on the selected binding residues of (A) *15:01 allele and (B) *16:01 allele.

Starting from the initial structure adopted for the production run, we have calculated the backbone root mean square deviation (RMSD) of the residues forming the binding site of the antigen/peptide MHC class II complex (chain α1 5–76 and chain β1 5–90) during 100 ns of MD simulations, performed in presence and absence of the two peptides (MBP, EBNA-1), as shown in Fig. 4. It is interesting to note that for the *15:01 allele (Fig. 4), the binding site displays a lower RMSD value when both peptide are bound (for MBP peptide ∼1.2 Å and for EBNA-1 peptide ∼1.7 Å), with respect to unbound case (∼3.1 Å). On the other hand for protective allele *16:01 (Fig. 4) the binding site in presence of MBP peptide displays a low average RMSD value (∼1.5 Å), followed by EBNA-1 (∼2.4 Å) and the unbound or no-peptide case (∼2.6 Å).

**Figure 5:**
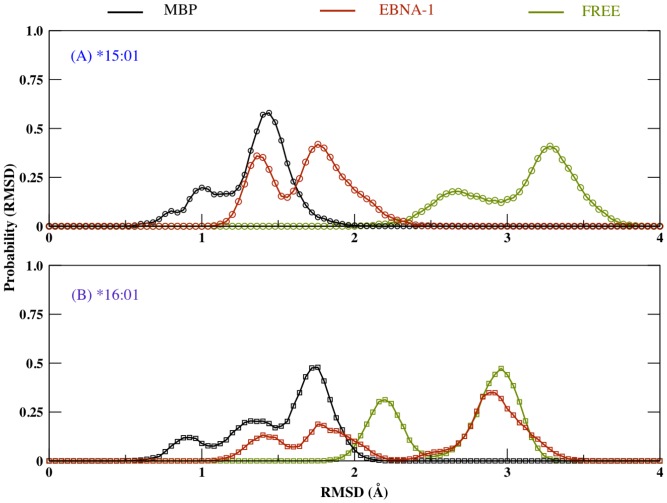
RMSD distribution plot. Probability distribution on C-alpha RMSD values obtained from MD simulations, on the selected binding residues of (A) *15:01 allele and (B) *16:01 allele.

In Fig. 5 we show the binding site RMSD distribution observed during the simulation. For MBP complexed with either of the alleles, binding site displays very similar RMSD distributions, while EBNA-1 displays a rather different RMSD pattern depending on the specific allele it is bound to (see Fig. 5A-B). These results suggest a higher binding site flexibility of *16:01 with respect to *15:01. Concerning the simulation of *16:01 unbound allele (Fig. 5B) we observe that the binding site RMSD distribution separates in two rather distinct distribution (the first with 30% probability and lower RMSD value ∼2.2 Å, while the second state with 50% probability and a high RMSD value ∼3.0 Å). On the contrary the *15:01 unbound allele (Fig. 5A), does not show such a net RMSD distribution split, but still exhibiting two peaks at 2.7 Å and 3.2 Å. These results suggest a higher flexibility of the binding site in the case of unbound predisposing allele (Fig. 5A). In summary, both average RMSD and RMSD distribution suggest a higher flexibility of the binding site in absence of peptide, thus confirming previous observations [51].

To quantify the flexibility of the binding site, the configurational entropy was calculated on carbon-alpha atoms of the binding site residues for both the alleles and in both free and bound cases. In Table 2, we have summarized the configurational entropy values obtained in the different cases. As expected, from the previous RMSD calculations, we observe binding site to posses the highest entropy in the simulations without peptide. On comparing the two alleles in either bound or unbound cases, we observe that the binding site of protective allele *16:01 to possess a higher entropy values (Table 2). Next, while comparing the two peptides, we find the binding site to exhibit higher entropy values when bound to non-self peptide EBNA-1 in both the alleles, thereby reflecting a higher flexibility.

**Table 2:** Configurational entropies.

	T▵S (kcal/mol)		
	MBP	EBNA-1	Free
**Predisposing**	1046	1060	1073
**Protective**	1060	1075	1083

Entropy contributions to the free energies calculated on binding site residues for the free and bound MHC.

### 2. Binding Groove dissection analysis

The binding groove of the HLA class II is approximately ∼40 Å long and is further characterized by a variable transversal width along its length (Fig. 3B). With the purpose to understand binding site flexibility on a local scale, the extended binding groove was divided into four compartments D1–D4, as similarly done previously [51].

#### Predisposing allele DRB1*15:01

**Figure 6:**
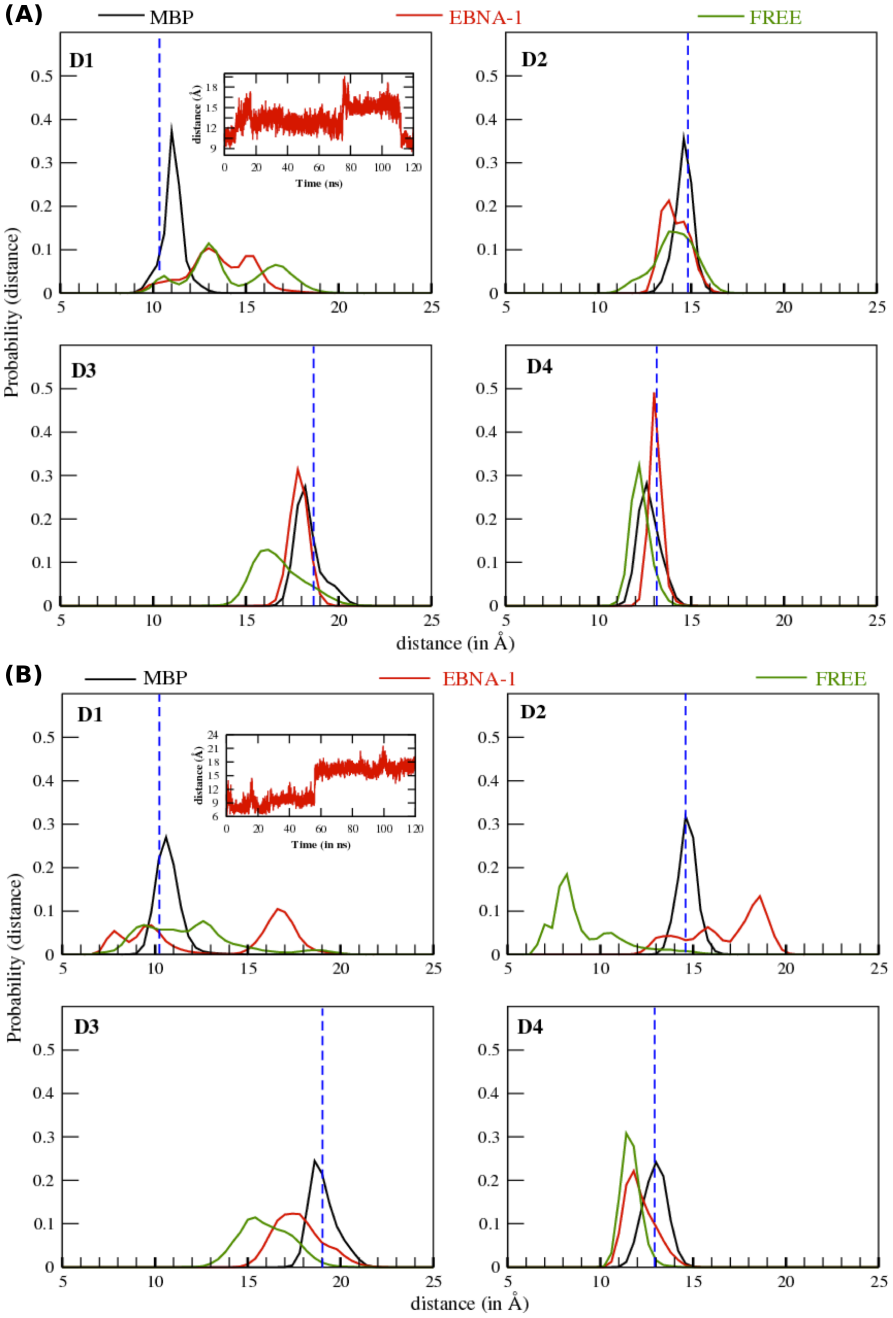
Local binding groove distance distribution plot. Normalized probability distribution of center of mass distance of heavy atoms between the flanking residues of chain A and chain B along different sections of binding groove (D1, D2, D3 and D4), for (A) *15:01 allele and (B) *16:01 allele.

When complexed with MBP, all the four regions of binding site exhibit an unimodal (single peak) distribution and with peak values close to their respective original X-ray structure values (Fig. 6A). When complexed with EBNA-1, the regions D3 and D4 of the binding site displays a unimodal distribution with peak values close to the X-ray structure values, while in region D1, binding site displays a complex distribution with a flat region around the width value of the X-ray structure (10.2 Å) and two almost equally probable peaks at 12.5 Å and 15 Å. In D2, binding site displays a bimodal distribution with two peaks at 13.5 Å and 14.5 Å, with the latter being close to the X-ray structure value. Finally, for the free MHC we note a significant distribution differences in the regions D1 and D3 with respect to the complexed peptide cases. In particular, in region D1 we observe a three peaked distribution (10.3 Å, 13 Å, 16.5 Å), with different probability and with the first value closer to the X-ray structure value, while in region D3 we observe a wide asymmetric unimodal distribution of ∼6 Å width and with a peak at ∼16 Å, which is less than the X-ray structure value (18.9 Å). To summarize, we observe that i) all the four regions of the binding site in presence of MBP are relatively narrower and show a width closer to the X-ray structure values, ii) while in the presence of EBNA-1, region D1 and D2 are more flexible (i.e display a wider distribution) with respect to case with MBP, and iii) finally in the free binding site, the regions D1, D2 and D3 are very flexible, as reflected by the wider distance distribution. Interestingly, region D4 in all the three cases displays quite a similar distance distribution.

#### Protective allele DRB1*16:01

When complexed with MBP, we observed all the four regions of the binding site to display a unimodal distribution (Fig. 6B) and with peak values close to the originally modeled structure of the allele, in a similar manner as observed for the predisposing allele. When the allele is bound to EBNA-1 peptide, we observe for D1 a three peaked distribution with peak values 7.5 Å, 9.5 Å and 16.5 Å, a flat region (∼12–15 Å) followed by two peaks at 15.5 Å and 18.5 in D2. Next, we observe a unimodal distribution in region D3 (ranging between 15 Å and 21 Å) and D4 (ranging between 10 Å and 15.0 Å). Finally, for the simulations without peptide, in D1 we observe a distribution with a plateau separating two peaks at 9.0 Å and 12.5 Å, in D2 a three peaked distribution with values 7.0 Å, 8.0 Å and 10.5 Å, and a unimodal distribution in D3 (ranging between 13 Å and 18 Å) and a narrow distribution in D4 (ranging between 11 Å and 13Å). In summary, the regions D1 and D2 exhibited more than one state for the EBNA-1 and the unbound cases, while a single state is present with MBP peptide. This evidence suggests a higher flexibility in the two regions (D1, D2) when HLA is unbound and bound with EBNA-1 peptide (Fig. 6B). On the other hand, region D3 and D4 exhibited very similar structural and dynamical behavior for the protective free or bound with EBNA-1 peptide, with both regions being narrower with respect to the MBP bound case, and more flexible in the D3 region (Fig. 6B).

### 3. Nature of Interactions

#### H-bond interactions

**Figure 7:**
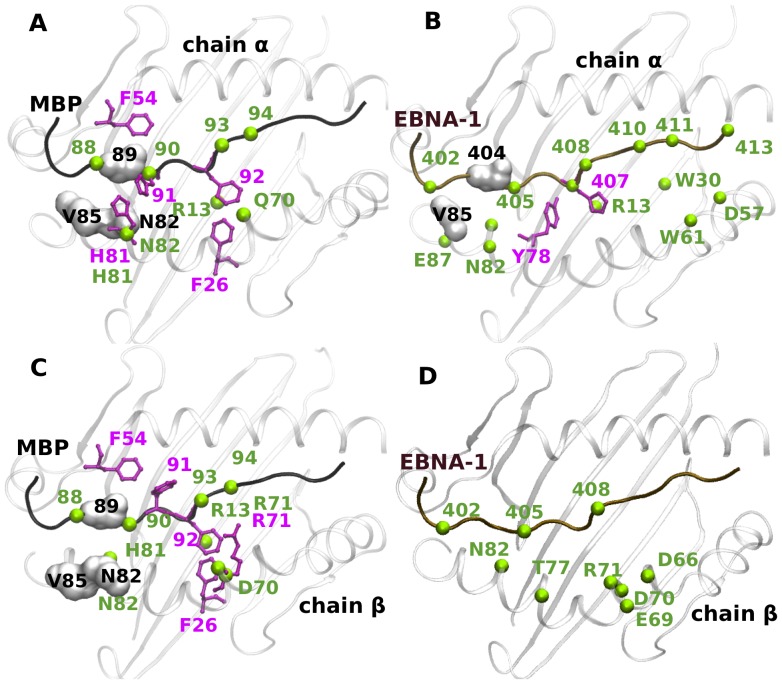
MHC-peptide Interaction Network. Persistent H-bonds (in green, ball representation), hydrophobic (in grey, surf representation) and stacking (in pink, ball-stick representation) interactions present during MD simulations for (A)*15:01-MBP (B)*15:01-EBNA-1 (C) *16:01-MBP and (D)*16:01-EBNA-1 complexes.

**Figure 8:**
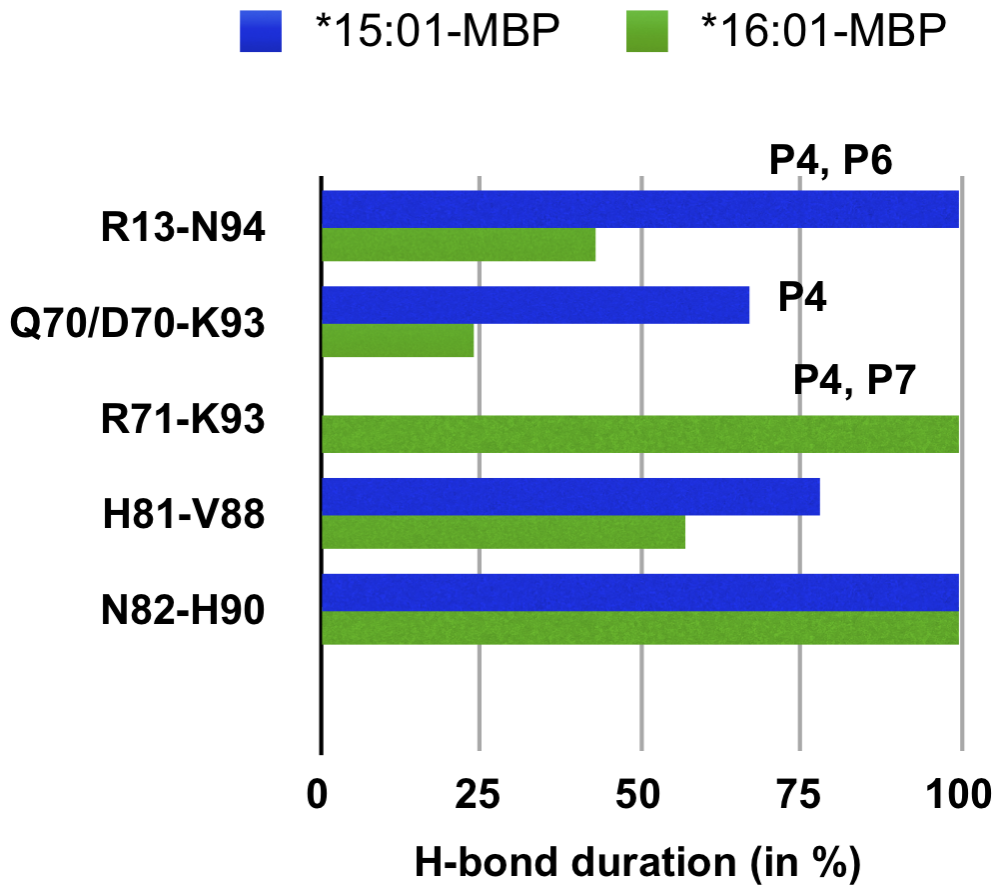
MBP-MHC H-bonds. Persistence of hydrogen bonds formed between MBP 85–98 peptide and the chain β1 5–90 residues of allele *15:01 (in blue) and *16:01 (in green), during long MD simulation of 120 ns.

**Figure 9:**
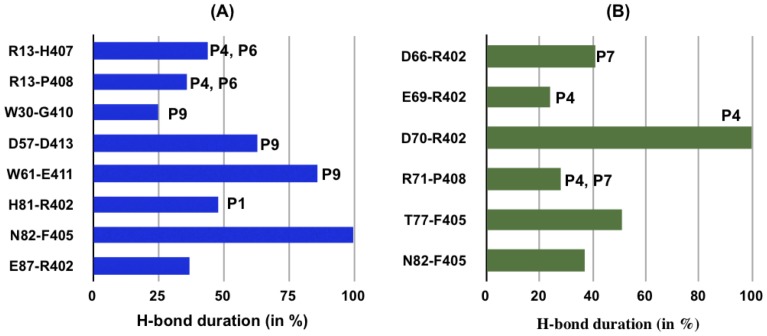
EBNA-1-MHC H-bonds. Persistence of hydrogen bonds formed between EBNA-1 400–413 peptide and the chain β1 5–90 residues of (A) *15:01 allele and (B) *16:01 allele, during long MD simulation of 120 ns.

We have also evaluated the H-bond interactions between the peptides (MBP, EBNA-1) and the binding site residues of both DRB1*15:01 and DRB1*16:01 alleles, present for at least 20% of simulation time (Fig. 7). First, from the peptide perspective, we observed MBP to make a similar network of interaction with both alleles (Fig. 7A, Fig. 7C), with the only notable exception of the interaction between residue Arg 71 (pocket 4) and MBP Lys 93, present only for the protective allele (Fig. 8). Even though we observe almost identical interacting pairs for alleles *15:01 and *16:01 complexed with MBP, the H-bond interactions in pocket 4 and pocket 6 are more durable in the case of the predisposing allele (Fig. 8). However, concerning the EBNA-1 peptide (Fig. 7B, Fig. 7D), we find a different network of interaction, with only one conserved interaction of binding site residue Asn 82 with EBNA-1 Phe 405 (Fig. 9). Secondly, from the allele perspective we observe that for the predisposing *15:01 allele, we find three residues of the binding site (Arg 13, His 81 and Asn 82) involved in interaction with both the peptides (Fig. 7A-B, Fig. 8, Fig. 9A). For the protective allele *16:01, we find three binding site residues (Asp 70, Arg 71 and Asn 82) involved in interaction with both peptides (Fig. 7, Fig. 8) with only one interacting pair (Asn 82-Phe 405) conserved with respect to the predisposing allele. Moreover, concerning the EBNA-1 complexes, we observed that H-bond interactions target pockets P4, P6 and P9 in the predisposing allele, while pockets P4, P7 in the protecting one.

#### Aromatic stacking and Hydrophobic Interaction

The stacking interactions were calculated between the peptide residues and the chain β1 of the binding site residues for the two alleles, which were present for at least for 20% of simulation time. In Table 3 we have summarized the observed stacking interaction during the MD simulations. In particular, for MBP we found that (i) residue Phe 92 is involved in stacking interaction with the β1-chain binding site residues Phe 26, Arg 71 of the protective allele (Fig. 7C), while only with Phe 26 in the predisposing allele (Fig. 7A), and (ii) MBP residue His 90 is involved in stacking interaction with only His 81 residue of predisposing allele (Fig. 7A). On the other hand, for peptide EBNA-1, we found residue His 407 to be involved in stacking interaction with the β1-chain residue Tyr 78 of the predisposing allele alone (Fig. 7B).

**Table 3:** Persistent aromatic stacking and hydrophobic interactions.

	Aromatic Stacking Interaction	Hydrophobic Interaction
**1501-MBP**	αF54-F91, βF26-F92, βH81-H90	βN82-V89, βV85-V89
**1501-EBNA-1**	βY78-H407	βV85-P404
**1601-MBP**	αF54-F91, βF26-F92, βR71-F92	βN82-V89, βV85-V89
**1601-EBNA-1**	Absent	Absent

The stacking and hydrophobic interaction for the bound and free MHC-peptide are reported below. The interacting pair is given as allele residue - peptide residue.

Next, we evaluated hydrophobic interactions between the binding site residues of chain β and the peptide residues, present for at least 20% of simulation time and without water molecules in a residue neighborhood of 4.0 Å (Table 3). We found hydrophobic interactions between MBP residue Val 89 with β-chain residues Asn 82 and Val 85 to be present for both alleles (Fig. 7A, Fig. 7C), while only EBNA-1 residue Pro 404 interacts with the β-chain residue Val 85 in the predisposing allele (Fig. 7B).

### 4. Binding Free Energy Calculation

The binding free energy for the MHC-peptide complexes was estimated using the solvated interaction energy method (see Material and Methods). MBP peptide complexed with predisposing allele displays very similar binding free energy value (−16.8 kcal/mol) with respect to EBNA-1 (−16.0 kcal/mol). In summary (Table 4A), we observe the binding energy of the predisposing allele in complex with both peptides displays quite similar values, while we note a significant difference in binding energy values between the self (MBP) and non-self (EBNA-1) peptides when complexed with the protective allele (Table 4B). More precisely, we observe a striking difference of ∼5 kcal/mol in the free energies between the MBP complex (−17.5 kcal/mol) and the EBNA-1 complex (−12.2 kcal/mol), confirming a higher stability of MBP complex, in accord to our previous analysis. To allow a better comparison between the data, the IC_50_ values obtained from binding energy values (see Material and Methods) for each allele were further transformed into a ratio relative to the MBP peptide (Table 4). Interestingly, for the protective allele (Table 4B) we note a very high IC_50_ ratio, which suggests a much weaker binding affinity of EBNA-1 (∼5×10^3^ times) for MHC compared to that of MBP peptide.

**Table 4:** Binding free energies calculations for peptide-MHC complexes.

	▵G (kcal/mol)	IC_50_ (nM)	IC_50_ ·/· IC_50__(MBP)_
*15:01-MBP	−16.7±0.6	5.2×10^−3^	1
*15:01-EBNA-1	−16.0±0.7	1.7×10^−3^	3
*16:01-MBP	−17.5±0.7	4.6×10^−4^	1
*16:01-EBNA-1	−12.2±0.7	2.5	5434

Predisposing (*15:01) and protective (*16:01) alleles bound to MBP and EBNA-1. In column 2 is reported the binding free energies (kcal/mol), in column 3 is reported their corresponding IC_50_ values and in column 4 is reported the IC_50_ scaled by the allele specific MBP IC_50_.

Next, we performed virtual single amino-acid substitution to Alanine, for MBP residues at the positions 89 to 94, since these positions were suggested to be important in either peptide-MHC binding (positions 89 and 92) or as TCR contact residues (positions 90, 91 and 93), as described in previous work [31], [73]. For EBNA-1 peptide we performed virtual single amino-acid substitution to alanine at positions 402–407, as this region was suggested to be important with regard to MS [32] and repeated the binding energy calculation for both the alleles. The calculated binding energies are summarized in Table S1 (supplementary material). In general, Ala substitution of peptide residues showed only a small reduction in the binding energy values with respect to non-mutated peptide, with a maximum reduction of 1 kcal/mol. Subsequently, we also performed Ala mutation on few selected MHC residues [19], [74] and re-evaluated the binding energy for the two peptide complexes with the two alleles (Table S2). As for mutation of peptide residues, we noted a small reduction in the binding energy values for the MHC mutated cases. The most significant change (∼1.2–1.5 kcal/mol) in the binding free energy is noted for α-chain residue at position 11 (Glu 11) mutated to Ala in both the alleles.

## Discussion

Long lived peptide-MHC complexes allow longer time span of antigen presentation, which is critical for the relatively slow process of recruiting specific antigen reactive T cells [75]. Furthermore, an optimal peptide-MHC flexible conformation has been associated to an improved TCR recognition [11], [13], [76], [77]. Therefore, in this study, we have performed long MD simulations (∼120 ns) for the two DR2 alleles (predisposing DRB1*15:01 and protective DRB1*16:01), free and complexed with the two relevant self (MBP) and non-self (EBNA-1) peptides, with the goal to determine the physicochemical properties governing MHC ability to bind antigenic peptides, that could also furnish information on efficacy of the antigen presentation to the T cell receptors. The RMSD analysis for the binding site residues shows that the binding groove is more stable only when complexed with a peptide (Fig. 4), consistent with previous MD simulations [8], [16], [19], [51]. Interestingly, comparing the bound MHC's, we find the binding site of the protective allele to be more flexible in particular when bound to non-self peptide (EBNA-1). Subsequently, concerning average RMSD alone, we can thus observe that, as expected for biological reasons, the presence of the peptide definitely stabilizes the MHC protein for both *15:01 and *16:01 alleles. Moreover, there is almost no RMSD distinction between the two peptides for the predisposing allele *15:01 (Fig. 4). On the contrary, the protective allele *16:01 shows a higher global flexibility in the presence of the pathogen-derived peptide (EBNA-1), very similar to that observed for the unbound allele (Fig. 4), suggesting a weak binding of the non-self antigen. Thus, we can postulate the higher flexibility shown by the unbound predisposing allele (Fig. 4) can facilitate its capability to accommodate both self (MBP) and non-self (EBNA-1) peptides in a similar manner and with similar final flexibility characteristics. On the contrary, the relatively higher rigidity of the unbound protective allele (Fig. 4) results in a less flexible binding site, incapable to bind both the peptides with similar affinities, in particular resulting in a less stable complex with the pathogen-derived peptide (EBNA-1). Subsequently, in the former case (predisposing) we can postulate a higher degree of functional and molecular mimicry between the self and the non-self peptide, thus leading to a higher possibility of T cell cross-reactivity [12], [78], with potential autoimmune consequences. This hypothesis could contribute to explain permissiveness of the allele with respect to MS. In fact, observing the RMSD probability distribution (Fig. 5A), we note a common peak, that is a common MHC configurational state for both peptides bound to *15:01 at ∼1.4 Å and a second less relevant common peak at ∼0.7 Å. To further investigate the flexibility origin with respect to the five binding pockets (Fig. 3A) traditionally defined in the binding site [45], we divided the binding groove into four compartments (Fig. 3B), covering most of the peptide-binding pockets and analyzed the structural changes in each one, for the free and bound alleles (Fig. 6). Interestingly, for the predisposing allele in region D1, we also observe the largest difference between the MBP and EBNA-1 distributions. In detail, in region D1, EBNA-1 complex and the unbound allele show a perfect overlap around 13 Å, while the MBP complex and the unbound allele exhibit distribution centered around ∼10–11 Å. These observations suggest the ability of the peptides to select two distinct configurations, out of the three possible ones offered by the unbound HLA protein. We can therefore hypothesize these three configurations correspond to (a) MBP peptide receptive state (∼10–11 Å) (b) EBNA-1 peptide receptive state (∼13.0 Å) and (c) unbound receptive state (∼17.0 Å). Moreover, we observe MBP and EBNA-1 distributions for the predisposing allele having an almost perfect overlap in region D3 at ∼17.5 Å, and a weaker overlap in region D2 at ∼14.5 Å, confirming the suggestions provided by the average RMSD (Fig. 4) and the RMSD distribution plots (Fig. 5), and localizing the source of possible structural and functional mimicry between the two peptides in the D3 region and to a less extent in the D2 region. Furthermore, for the unbound predisposing allele we observed a closure in region D3 with respect to the bound allele. Notably, this closure corresponds to an energetically favored extended conformation, caused by re-arrangement of residues in the D3 region and intra-protein H-bonds, as also suggested in a previous MD study [19]. Concerning the protective allele, the closure in the D2 region is facilitated by two intra-chain H-bond interactions (α Ser 53 - β Asn 82 and α Glu 55 - β Asn 82). As mentioned for the predisposing HLA, we observe a similar cleft closure in region D3 for the unbound protective allele.

Polymorphic residues at position 70, 71, and 74 (pocket 4) in the DR β chain -known as restrictive super-type patterns- have been linked with susceptibility or resistance to autoimmune diseases [79], with the allele *15:01 and *16:01 possessing “QAA” and “DRA” pattern, respectively. Interestingly, the difference between these two patterns is nicely reflected in the nature of the interaction between the peptide and the associated MS alleles also in the present study (Fig. 7). For instance, in the MBP bound cases, the only notable difference in H-bond interaction network between protective and predisposing alleles is due to a polymorphism at position 71 (Fig. 8). In the EBNA-1 bound cases, in addition to an MBP-like trend at position 71, we observe a polymorphism also at position 70: Gln in the predisposing allele and Asp in the protective one. Furthermore, the same polymorphism at position 71 is the reason of different aromatic stacking interaction networks in the two alleles complexed with MBP (Fig. 7A, Fig. 7C, Table 3). Previous experimental studies [80] have shown that a conserved residue in both alleles at polymorphic position 13 (Arg 13, pocket 4 and 6) is one of the few key amino acids known to be important for antigen binding and potentially relevant to MS. This aspect is also confirmed in the present study, where Arg 13 is found to be involved in durable H-bond interaction with MBP in both the alleles (Fig. 8), while it is involved in the predisposing allele in complex with only EBNA-1 (Fig. 9A).

## Conclusions

On analyzing the interaction network featured by the two peptides when bound alternatively to the two alleles (Fig. 7), we find the protective allele to exhibit significant specific binding properties characterizing the MBP and EBNA-1 peptide complexes (Fig. 7C-D). Our finding was further confirmed by calculating the binding free energies of peptide-protective allele complex that provide a good way to capture the stability of peptide-MHC complex, which is essential for successful peptide recognition by the T cell receptor. In particular, we noted a significant difference of ∼5 kcal/mol between the MBP and EBNA-1 peptide bound cases (Table 4B). The interaction characteristics and binding energies obtained in our study support a molecular functional mimicry between the peptides MBP and EBNA-1 when complexed with the predisposing allele. Furthermore, in our study we were also able to demonstrate similar functional behavior of the two alleles in binding MBP, in accordance to previous experimental findings [81].

In summary, while the predisposing allele exhibits a coherently conserved interaction network with the self (MBP) and non-self (EBNA-1) peptides (Fig. 7A-B), the protective allele is capable to discriminate the two peptides (Fig. 7C-D) and possesses unique stacking and hydrophobic interactions with MBP peptide alone. We have also demonstrated how new and “classically” observed residues and motifs contributions to the predisposing allele nature can be explained at a molecular level in terms of interaction networks, conferring to the allele specific dynamical characteristics when interacting with distinct peptides. In conclusion, this study addressed the structural and dynamical comparison of the two MS disease relevant alleles in Sardinian population, highlighting their different binding characteristics together with an analysis of their physicochemical properties. Due to very limited or no experimental data available for the EBNA-1-MHC complexes investigated in the current study, our computational results were not corroborated directly by experiments. In any case, our findings could help to design binding assays for the MS susceptible alleles and their specifically associated epitopes, subject of the present study, on the same path adopted in previous works [21], [81]. However, we support our computational findings by comparing them to similar molecular simulations and related experiments, in an attempt to clarify potential immunological significance of our outcomes with respect to multiple sclerosis. We believe the presented approach would assist in understanding the molecular basis of the disease and could further be translated to experiments and clinical applications, including therapeutic peptide design to modulate peptide-MHC affinity [82].

## Supporting Information

Table S1 **Binding free energies for peptide mutations.** Free binding energies (kcal/mol) are reported for peptide- MHC complexes with mutation to Alanine for (A) MBP at residue positions 89 to 94, and (B) EBNA-1 at residue positions 402 to 407.
(DOC)

Table S2 **Binding free energies on selected MHC mutations.** Differences in binding free energies (kcal/mol) between bound MHC in native and on Alanine mutation for selected residues of β-chain and α-chain residues**.**
(DOC)
