# Supplementary material for: Structural and Dynamical Insights on HLA-DR2 Complexes That Confer Susceptibility to Multiple Sclerosis in Sardinia: A Molecular Dynamics Simulation Study
Source: PLoS One. 2013 Mar 26;8(3):e59711. doi: 10.1371/journal.pone.0059711 (PMC3608583; doi:10.1371/journal.pone.0059711)
Supplement: Table S1 — Binding free energies for peptide mutations. Free binding energies (kcal/mol) are reported for peptide- MHC complexes with mutation to Alanine for (A) MBP at residue positions 89 to 94, and (B) EBNA-1 at residue positions 402 to 407. (DOC) [file pone.0059711.s001.doc]

(A) MBP

|  | **V89A** | **H90A** | **F91A** | **F92A** | **K93A** | **N94A** |
| --- | --- | --- | --- | --- | --- | --- |
| ***15:01** | -16.1±0.6 | -16.4±0.6 | -15.9±0.6 | -15.8±0.6 | -16.3±0.6 | -15.6±0.6 |
| ***16:01** | -16.9±0.6 | -17.1±0.6 | -16.7±0.6 | -16.3±0.6 | -17.2±0.6 | -16.4±0.6 |

(B) EBNA-1

|  | **R402A** | **R403A** | **P404A** | **F405A** | **F406A** | **H407A** |
| --- | --- | --- | --- | --- | --- | --- |
| ***15:01** | -15.1±0.7 | -15.1±0.7 | -15.5±0.7 | -15.6±0.7 | -14.9±0.7 | -15.3±0.7 |
| ***16:01** | -11.6±0.7 | -11.2±0.7 | -12.2±0.7 | -11.6±0.7 | -11.7±0.7 | -11.5±0.7 |
