# Supplementary material for: Structural and Dynamical Insights on HLA-DR2 Complexes That Confer Susceptibility to Multiple Sclerosis in Sardinia: A Molecular Dynamics Simulation Study
Source: PLoS One. 2013 Mar 26;8(3):e59711. doi: 10.1371/journal.pone.0059711 (PMC3608583; doi:10.1371/journal.pone.0059711)
Supplement: Table S2 — Binding free energies on selected MHC mutations. Differences in binding free energies (kcal/mol) between bound MHC in native and on Alanine mutation for selected residues of β-chain and α-chain residues. (DOC) [file pone.0059711.s002.doc]

|  | **∆G (wild type) - ∆G (mutant)** | | | |
| --- | --- | --- | --- | --- |
|  | **MBP** | | **EBNA-1** | |
|  | ***15:01** | ***16:01** | ***15:01** | ***16:01** |
| **βN82A** | 0.8 | 0.4 | 0.5 | 0 |
| **βR71A** | x | 0.7 | x | 0.3 |
| **αF54A** | 0.5 | 0.4 | 0.6 | 0.3 |
| **αE11A** | 1.2 | 1.5 | 0.5 | 0.6 |
